# Processing speed moderates the relationship between age and crystallized intelligence and influences the indirect effect of age on verbal working memory in middle-aged and older adults

**DOI:** 10.3389/fnagi.2026.1736609

**Published:** 2026-04-10

**Authors:** Julio Menor

**Affiliations:** Department of Psychology, University of Oviedo, Oviedo, Spain

**Keywords:** cognitive aging, crystallized abilities, fluid abilities, processing speed, working memory and aging, moderated mediation model

## Abstract

This study examined the extent to which processing speed moderates the relationship between age and crystallized intelligence and influences the indirect effect of age on verbal working memory in 233 healthy middle and older adults aged 51–94 years. Participants were screened for cognitive impairment and depressive symptoms before completing a battery of cognitive tests. Data were analyzed using moderated mediation models with bootstrapping. Correlational analyses revealed significant negative associations between age and all cognitive variables. Processing speed showed the strongest negative correlation with age, while crystallized intelligence and working memory also declined with advancing age. Strong positive correlations were observed among the three cognitive indices. In the first moderated mediation analysis, processing speed moderated the relationship between age and crystallized intelligence. Specifically, age was associated with higher crystallized intelligence only among participants with high processing speed, which in turn predicted better working memory. No such effect was observed for individuals with average or low processing speed. In the second analysis, crystallized intelligence did not moderate the link between age and processing speed, suggesting differential effects. These findings indicate that preserved processing speed enables older adults to maintain crystallized knowledge, which supports working memory. Conversely, declines in processing speed may limit the protective role of crystallized abilities. Although constrained by its cross-sectional design, the study underscores the interactive roles of fluid and crystallized abilities in cognitive aging and identifies potential targets for cognitive training interventions.

## Introduction

With advancing age, healthy adults typically show declining performance across many different domains of cognitive functioning. Age-related cognitive declines begin to emerge as early as middle adulthood, occur fairly continuously over time, affect individuals without diagnosed pathologies, and are observed across the full range of psychological and physical health (Salthouse, 2004, 2009). Results from cross-sectional and quasi-longitudinal comparisons indicate that normative cognitive aging is characterized by nearly linear declines in processing speed from early adulthood, and accelerating declines in working memory, episodic memory, and reasoning. However, vocabulary knowledge continues to increase until the 60s (Salthouse, 2019). Healthy aging has been associated with reductions in gray matter volume and functional alterations in several regions that are crucial for higher cognitive function (Loaiza, 2024; Prince et al., 2024). The age-related cognitive differentiation/dedifferentiation hypothesis suggests that the functional organization of intellectual abilities is relatively compressed in childhood, undergoes differentiation during maturation, and becomes dedifferentiated again in old age (e.g., Baltes et al., 1980, 1998; Breit et al., 2022), leading to a reduction in the specificity of neural representations and potentially impacting cognitive performance. As people age, the brain’s functional organization becomes less specialized, and different cognitive processes increasingly rely on overlapping neural networks (Prince et al., 2024).

The classic fluid (mechanics)–crystallized (pragmatics) model of individual differences in cognitive functioning distinguishes between two broad classes of abilities (Horn, 1989). Crystallized abilities refer to the use of skills, knowledge, and experience acquired through life, culture, and education (Carroll, 1993). They are assessed through vocabulary tests, general knowledge questions, domain-specific skills, and problem-solving tasks based on prior knowledge. Crystallized abilities increase throughout adulthood and tend to remain stable or decline later in life (Li et al., 2004; Salthouse, 2006). These abilities have been considered indicators of cognitive reserve (Tucker-Drob et al., 2009; Tucker-Drob, 2011, 2019) and can contribute to it by providing a base of knowledge and skills that can help buffer against cognitive decline. Fluid abilities, in contrast, require effortful processing at the time of assessment and encompass domains such as perceptual speed, working memory, abstract reasoning, and visuospatial reasoning (Horn, 1989). Declines in fluid abilities particularly affect complex or novel tasks requiring active processing (Zacks et al., 2000), raising the question of how older adults cope with daily decision-making. Different studies have shown that fluid and crystallized abilities show diverging average trajectories across adulthood: fluid abilities progressively decline from early to middle adulthood, whereas crystallized abilities continue to increase until around the seventh decade of life (Lindenberger, 2001; Rönnlund et al., 2005; Schaie, 2005). This divergence has been interpreted as evidence for a compensatory mechanism in which gains in crystallized abilities help offset fluid losses, thereby prolonging independent functioning into late adulthood. However, Tucker-Drob et al. (2022), using independent longitudinal datasets, found that rates of change are strongly correlated across both fluid and crystallized abilities. Their results show that individuals who experience greater losses in fluid abilities tend to show smaller gains—or even losses—in crystallized abilities, suggesting that the two domains become increasingly interrelated rather than compensatory.

Two important domains of fluid abilities are working memory (WM) and processing speed. WM enables temporary storage and manipulation of information, integrating knowledge retrieved from long-term memory (Baddeley et al., 2021). It is essential for higher-order cognitive processes and for performing complex everyday tasks (Engle, 2002; Oberauer, 2009). For example, it allows one to remember the order of steps in a recipe while cooking and to keep several options in mind while making a decision (Labra Pérez and Menor, 2018). Further, reduced WM is associated with a decrease of older adults’ autonomy and presonal wellbeing (Klingberg, 2012). A large body of literature has established that WM generally declines in old age (e.g., Bopp and Verhaeghen, 2005; Borella et al., 2008; Chai et al., 2018; Rhodes and Katz, 2017; Sander et al., 2012). Although most individuals experience a gradual decline of their working memory with advancing age, some older adults preserve their working memory abilities, and others manifest a sharp decline (Cansino et al., 2020). However, it remains unclear what factors moderate this decline. One potential factor is crystallized ability—such as educational attainment, which partly reflects the breadth of knowledge stored in long-term memory. Yet findings are inconclusive. Some early studies suggested that higher education attenuates age-related declines in WM performance (e.g., Bosma et al., 2003), but later research failed to confirm this. For example, in the Victoria Longitudinal Study (Zahodne et al., 2011), although years of education were strongly associated with WM performance, they did not predict rates of decline. Comparable patterns have been found for cognitive reserve, which appears to benefit both younger and older adults (Speer and Soldan, 2015). Cansino et al. (2020) analyzed the predictors of working memory performance in an N-back task in healthy adults between 61 and 80 years of age. They found that WM decline was predicted by lower scores on the vocabulary test and in those who reported more depressive symptoms. Vocabulary scale scores were also significant predictors of working memory maintenance. Thus, vocabulary scale scores acted as both protective and risk factors of working memory, suggesting that they are not only relevant for successfully maintaining WM but also crucial for avoiding severe WM decline. Understanding the interplay between crystallized abilities (and cognitive reserve more broadly) and WM is crucial for explaining how cognitive health is maintained with age (Lojo-Seoane et al., 2020; Neubeck et al., 2022).

The second domain of fluid ability is processing speed, which refers to the rate at which individuals perceive, process, and respond to information, and is operationalized through tasks requiring rapid symbol substitution, reaction time, or perceptual comparisons (Salthouse, 1996). Processing speed is considered a fundamental cognitive capacity and has been regarded as a strong marker of cognitive decline across adulthood and old age (Salthouse, 1991; Salthouse et al., 1996; Verhaeghen and Salthouse, 1997). Individuals with higher processing speed tend to have greater WM capacity, particularly on complex tasks or those that involve multitasking (Fry and Hale, 1996; Bopp and Verhaeghen, 2005). Processing speed is also a major factor in the decline of WM, as it limits the time available for complex cognitive operations and for updating information (Salthouse, 1996, 2019).

The relationships among age, WM, crystallized intelligence, and processing speed are complex and dynamic, and the exact contribution of each mechanism remains uncertain (Fry and Hale, 1996; Naveh-Benjamin and Cowan, 2023; Neubeck et al., 2022; Tucker-Drob et al., 2022). Processing speed and knowledge may jointly influence WM performance. On one hand, the protective role of knowledge is important for WM during aging (Barulli and Stern, 2013). On the other hand, processing speed facilitates knowledge acquisition and accumulation (Ghisletta and Lindenberger, 2003), which are central to crystallized intelligence. Faster processing speed supports more efficient learning (e.g., acquiring new vocabulary and applying learned concepts more quickly), thereby enhancing crystallized intelligence. Indeed, Ghisletta and Lindenberger (2003) and Ghisletta and de Ribaupierre (2005) demonstrated that changes in crystallized abilities (e.g., vocabulary knowledge) are better predicted by changes in fluid abilities (e.g., processing speed) than the reverse.

The aim of the present study was to analyze the relationships between processing speed, crystallized abilities, and WM performance in healthy older adults using moderated mediation models. These models allow for the examination of conditional indirect effects, that is, identifying boundary conditions for a particular mechanism by testing how the strength of an indirect effect varies across subgroups or conditions (Hayes, 2018). Specifically, we analyzed the extent to which processing speed moderates the indirect effect of age on WM through crystallized intelligence. Age is expected to negatively affect WM; however, this effect is likely mediated by crystallized abilities, such that individuals with higher crystallized abilities show better WM performance, whereas those with lower crystallized abilities display poorer performance. Moreover, this relationship is anticipated to be moderated by processing speed, such that individuals with higher processing speed are better able to maintain the benefits of crystallized abilities, whereas those with lower processing speed will exhibit diminished benefits. However, considering the results of Ghisletta and Lindenberger (2003), crystallized abilities are not expected to moderate the relationship between age and processing speed.

## Materials and methods

### Participants

The initial sample consisted of 250 participants aged 51–94 years. They were screened using the Mini-Mental State Examination (M = 29.36, SD = 0.85; Folstein et al., 1975; Spanish version: Blesa et al., 2001) to exclude those with cognitive impairment, and the geriatric depression scale (GDS-30; Yesavage et al., 1982) to assess the presence of depressive symptoms. All participants lived at home and were recruited from leisure clubs and a university program for older adults. Exclusion criteria included a history of psychiatric or neurological disorders, head injury, symptoms of cognitive impairment (MMSE < 26) and the presence of depressive symptoms (GDS-30 scale > 10). Seventeen participants were excluded, sixteen due to depressive symptoms and one because of a diagnosis of epilepsy. The final sample consisted of 233 participants (155 women and 78 men). All participants provided informed consent in accordance with the Declaration of Helsinki. A *post hoc* sensitivity power analysis with G*Power (Faul et al., 2007) indicated that for this sample size, α = 0.05 and 1−β = 0.80, the minimum detectable effect size is *f*^2^ = 0.034. Furthermore, Monte Carlo simulations (Fritz and MacKinnon, 2007) indicate that a sample of 200–250 participants provide 80% power for detecting moderated indirect effects.

### Procedure and instruments

Assessments were conducted individually in a quiet room by a psychologist trained in test administration. The following tests were administered: The Mini-Mental State Examination (MMSE; Folstein et al., 1975), corrected for age and education according to the Spanish adaptation (Blesa et al., 2001), and the geriatric depression scale (GDS-30; Yesavage et al., 1982) were used for initial screening. Subsequently, the following WAIS-III subtests (Wechsler, 1999) were administered: the Vocabulary subtest for crystallized intelligence; the Symbol Search and Digit Symbol Coding subtests for processing speed; and the Arithmetic, Digit Span Forward, Digit Span Backward, and Letter–Number Sequencing subtests for verbal working memory. The Vocabulary subtest evaluates the individual’s verbal knowledge, word comprehension, and ability to retrieve and use words appropriately. Performance on this subtest reflects the development of language skills, as well as cultural, educational, and experiential influences, and provides an indication of long-term verbal memory (Wechsler, 1999). The Digit Symbol Coding subtest requires the examinee to use a key pairing digits with symbols and to reproduce the corresponding symbols for a sequence of numbers as quickly and accurately as possible. This task primarily assesses processing speed, visual-motor coordination, and attention to detail (Wechsler, 1999). The Symbol Search subtest measures visual perception, processing speed, and sustained attention. The examinee must rapidly determine whether a target symbol appears among a group of other symbols (Wechsler, 1999; Kaufman and Lichtenberger, 2006). Performance on the Digit Span subtest represents a combined measure of auditory attention, concentration, and working memory capacity, as assessed through both forward and backward recall conditions (Wechsler, 1999; Conway et al., 2003). The Arithmetic subtest consists of orally presented mathematical word problems that progressively increase in difficulty. Performance reflects mental computation ability, concentration, and working memory, as difficulty is determined by the amount of information that must be mentally retained and manipulated to arrive at the correct solution (Wechsler, 1999). Finally, the Letter-Number Sequencing subtest involves the oral presentation of mixed sequences of numbers and letters. The examinee is required to recall the sequence by first repeating the numbers in ascending order, followed by the letters in alphabetical order. This subtest primarily assesses working memory, attention, and sequencing abilities (Wechsler, 1999; Kaufman and Lichtenberger, 2006).

### Data analyses

Prior to conducting moderated mediation analyses, assumptions of independence of observations, linearity, multicollinearity, and normality on the raw scores were tested (Clement and Bradley-Garcia, 2022). The Durbin–Watson statistic was 1.79, indicating that the assumption of independence was met (acceptable range = 1.5–2.5; Glen, 2022). Variance inflation factor values were below 10 (Miles, 2005), indicating no multicollinearity. Normality was assessed using skewness and kurtosis statistics, calculated by dividing the estimates by their standard errors. Distributions with z scores between ± 3.29 were considered normal (see Table 1; Kim, 2013). The correlation between Vocabulary and years of education was 0.57 (*p* < 0.001). Correlations among Letter–Number Sequencing, Arithmetic, and Forward and Backward Digit Span were greater than 0.50 (all p_*s*_ < 0.001) and the correlation between Symbol Search and Digit Symbol Coding was 0.82 (*p* < 0.001). Negative and significant correlations were obtained between the GDS scale scores and the cognitive tests (all p_*s*_ < 0.01, see Table 1). Raw scores from the cognitive assessment tests were transformed into z scores. Composite indices were then calculated by averaging the z-scores: Processing Speed Index (Symbol Search and Digit Symbol Coding), Crystallized Intelligence Index (Vocabulary and years of education), and Verbal Working Memory Index (Letter–Number Sequencing, Arithmetic, Forward Digit Span, and Backward Digit Span). Correlations between age and the composite indices were subsequently computed. Descriptive statistics and correlations are shown in Tables 1, 2, respectively.

**TABLE 1:** Descriptive statistics for demographic and cognitive variables.

Demographic and cognitive variables	Mean	SD	Sk	Ratio Sk/SE	K	Ratio K/SE
Age	68.27	8.45	0.33 (0.16)	2.06	−0.30 (0.32)	−0.93
GDS	4.82	2.84	0.07 (0.16)	0.44	−1.0 (0.32)	−3.12
Years of education	10.76	4.47	0.54 (0.16)	3.37	0.43 (0.32)	1.34
Vocabulary	45.60	8.63	−0.34 (0.16)	−2.12	0.49 (0.32)	1.53
Digits: total score	13.03	3.10	0.57 (0.17)	3.35	0.56 (0.32)	1.75
Arithmetic	12.10	3.44	0.48 (0.16)	3.0	−0.66 (0.32)	−2.06
L-N sequencing	8.34	2.62	0.11 (0.16)	0.69	0.28 (0.32)	0.87
Symbol search	22.23	8.97	−0.04 (0.16)	−0.25	−0.45 (0.32)	−1.40
Digit symbol coding	45.10	18.1	0.44 (0.16)	2.75	−0.23 (0.32)	−0.71

SD, standard deviation; L-N sequencing, letters and numbers sequencing; GDS, geriatric depression scale; Sk, skewness; K, kurtosis; SE, standard error. Standard errors in brackets.

**TABLE 2:** Intercorrelations between age, cognitive variables and composite measures.

Cognitive and composite measures	2	3	4	5	6	7	8	9	10	11	12
1. Age	−0.16*	−0.30***	−0.36***	−0.32**	−0.31***	−0.50***	−0.60***	−0.60***	−0.37***	−0.63***	−0.45***
2. GDS		−0.18**	−0.28***	−0.23***	−0.27***	−0.17**	−0.22**	−0.28**	−0.26***	−0.26**	−0.27***
3. Years of education			0.57***	0.38***	0.44***	0.48***	0.53**	0.58***	0.89***	0.58***	0.51***
4. Vocabulary				0.47***	0.51***	0.53***	0.54**	0.58***	0.89***	0.59***	0.60***
5. Digits: total score					0.54***	0.60***	0.47**	0.50***	0.48***	0.51***	0.84***
6. Arithmetic						0.58***	0.53**	0.52***	0.54***	0.54***	0.84***
7. L-N sequencing							0.68**	0.65***	0.57***	0.69***	0.86***
8. Symbol search								0.82***	0.60***	0.95***	0.66***
9. Digit symbol coding									0.65***	0.96***	0.66***
10. Crystallized intelligence										0.66***	0.63***
11. Speed processing											0.69***
12. Working memory											

SD, standard deviation; L-N sequencing, letters and numbers sequencing; GDS, geriatric depression scale.

*p < 0.05,

**p < 0.01,

***p < 0.001.

**FIGURE 1:**
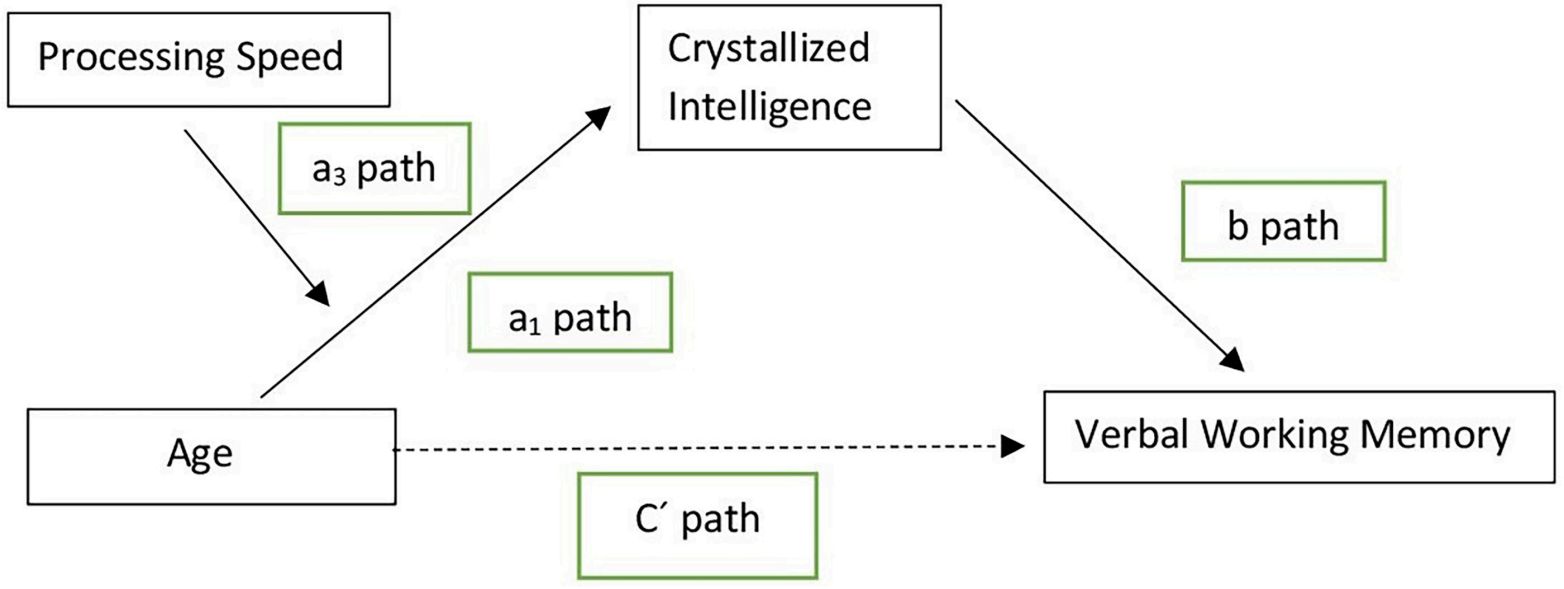
Hypothesized moderate mediation model.

The hypothesized moderated mediation model (see Figure 1) was tested using a bootstrapping approach to assess the significance of indirect effects at different levels of the moderator (Hayes, 2018). Two moderate mediation analyses were conducted. In the first, age was the predictor variable, crystallized intelligence the mediator, working memory the outcome, and processing speed the moderator. In the second, age was the predictor, processing speed the mediator, working memory the outcome, and crystallized intelligence the moderator. To control the influence of depressive symptoms, GDS scores were included as a covariate in both models.

Moderated mediation analyses test the conditional indirect effect of a moderator on the relationship between a predictor and an outcome via mediators. The PROCESS macro (model 7; Hayes, 2018) in SPSS v.27 was used, with 10,000 bias-corrected bootstrap samples and 95% confidence intervals. An index of moderate mediation was computed to test the significance of the effects. Significant effects were supported when zero was not included in the confidence intervals.

## Results

### Correlational analyses

Age and GDS scores correlated negatively with all cognitive assessment tests (all p_*s*_ < 0.01). Furthermore, the bivariate correlations between age and the indices of cognitive functioning were all significant and negative (all p_*s*_ < 0.001); that is, as age increased, the Working Memory Index, Processing Speed Index, and Crystallized Intelligence Index decreased. The correlations among the three cognitive indices were positive and exceeded 0.60, *p* < 0.001 (see Table 2).

### Analysis of moderate mediation: tests of conditional indirect effects

**FIGURE 2:**
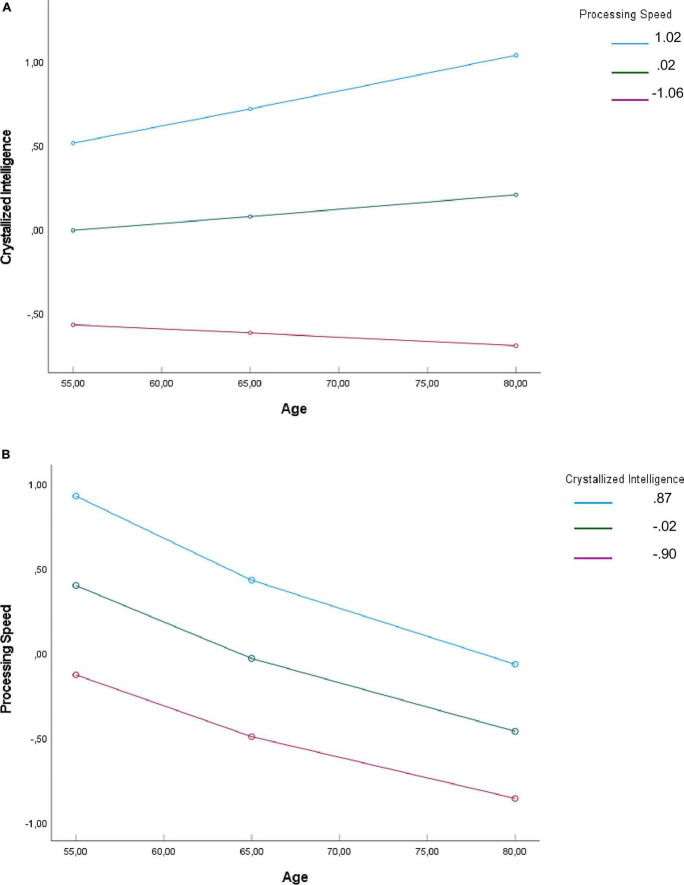
**(A)** Simple slopes of age predicting crystallized intelligence for 1 SD below the mean of processing speed, the mean of processing speed. **(B)** Simple slopes of age predicting processing speed for 1 SD below the mean of crystallized intelligence, the mean of crystallized intelligence, and 1 SD above the mean of crystallized intelligence.

First model (Table 3A and Figure 2A): Processing speed was found to moderate the effect of age on crystallized intelligence (unstandardized interaction B = 0.15, SE = 0.04, *t* = 3.32, *p* < 0.001). Tests of simple slopes (i.e., conditional effects on path *a*) revealed a significant association between age and crystallized intelligence for participants with high processing speed scores (B = 0.25, SE = 0.08, *t* = 3.18, *p* = 0.002), but not for those with medium (B = 0.10, SE = 0.05, *t* = 1.80, *p* = 0.07) or low scores (B = −0.06, SE = 0.06, *t* = −0.89, *p* = 0.37). When verbal working memory was considered as outcome the interaction between age and crystallized abilities was not significant [*F*(1, 226) = 0.53, *p* = 0.47] and greater crystallized abilities were associated with greater working memory (B = 0.49, SE = 0.05, *t* = 9.93, *p* < 0.001). Depressive symptoms were included as a covariate in the model. The results showed that depressive symptoms had a marginally significant direct effect on working memory performance. The overall moderated mediation model was supported, with the index of moderated mediation B = 0.074, 95% CI [0.03, 0.12]. Because zero was not within the CI, this indicates a significant moderating effect of processing speed on the indirect effect via crystallized abilities. Conditional indirect effects of age on working memory performance via crystallized intelligence were significant for high processing speed (indirect effect = 0.12, 95% CI [0.04, 0.21]), but not for medium (indirect effect = 0.05, 95% CI [−0.007, 0.11]) or low (indirect effect = −0.03, 95% CI [−0.08, 0.03]) processing speed. A significant direct effect of age on working memory remained after controlling for crystallized intelligence (B = −0.20, SE = 0.04, *t* = −4.45, *p* < 0.001, 95% CI [−0.29, −0.11]).

**TABLE 3A:** Unstandardized regression coefficients of the moderated mediation model for verbal working memory.

Tested model	B	SE	*t*	*p*	95% CI
Predicting the mediator (crystallized intelligence)					
Age (a path)	0.10	0.06	1.57	0.08	−0.01 0.21
Processing speed	0.68	0.06	11.76	< 0.001	0.56 0.79
Age X processing speed	0.15	0.04	3.32	< 0.001	0.06 0.24
GDS	−0.06	0.04	−1.36	0.18	−0.15 0.07
Predicting the outcome variable (verbal working memory)					
Age (c′ path)	−0.20	0.04	−4.57	< 0.001	−0.29 −0.12
Crystallized intelligence (b path)	0.49	0.05	9.93	< 0.001	0.40 0.61
GDS	−0.08	0.04	−1.95	0.052	−0.17 0.007
Conditional indirect effects for processing speed					
Low (1 SD below mean)	−0.03	0.03			−0.08 0.03
Mean	0.05	0.03			−0.006 0.11
High (1 SD above mean)	0.13	0.04			0.04 0.22

Mediator variable: crystallized intelligence. Moderator variable: processing speed. GDS, geriatric depression scale; CI, confidential interval.

Second model (Table 3B and Figure 2B): Crystallized intelligence did not moderate the effect of age on processing speed (unstandardized interaction B = −0.07, SE = 0.05, *t* = −1.48, *p* = 0.14). When verbal working memory was considered as outcome the interaction between age and processing speed was not significant [*F*(1, 226) = 0.95, *p* = 0.33] and greater processing speed was associated with greater working memory (B = 0.58, SE = 0.06, *t* = 10.35, *p* < 0.001). The index of moderated mediation was not significant (B = −0.04, 95% CI [−0.10, 0.01]). Because zero was within the CI, this indicates that the moderating effect of crystallized intelligence on the indirect effect via processing speed was not significant. The direct effect of age on verbal working memory after controlling for processing speed was not significant (B = −0.01, SE = 0.05, *t* = −0.24, *p* = 0.81, 95% CI [−0.11, 0.09]).

**TABLE 3B:** Unstandardized regression coefficients of the moderated mediation model for verbal working memory.

Tested model	B	SE	*t*	*p*	95% CI
Predicting the mediator (processing speed)					
Age (a path)	−0.43	0.04	−10.07	< 0.001	−0.51 −0.35
Crystallized intelligence	0.52	0.05	10.56	< 0.001	0.43 0.62
Age X crystalized intelligence	−0.07	0.05	−1.48	0.14	−0.17 0.02
GDS	−0.07	0.04	−1.81	0.07	−0.15 0.006
Predicting the outcome variable (verbal working memory)					
Age (c′ path)	−0.01	0.05	−0.24	0.81	−0.11 0.09
Processing speed (b path)	0.58	0.06	10.36	< 0.001	0.47 0.69
GDS	−0.07	0.04	−1.74	0.08	−0.15 0.01
Conditional indirect effects for crystallized intelligence					
Low (1 SD below mean)	−0.90	0.04			−0.29 −0.14
Mean	−0.02	0.03			−0.32 −0.18
High (1 SD above mean)	0.87	0.05			−0.38 −0.20

Mediator variable: processing speed. Moderator variable: crystallized intelligence. GDS, geriatric depression scale; CI, confidential interval.

## Discussion

The purpose of this study was to analyze the relationships among crystallized abilities, processing speed, and WM performance using moderated mediation models in older adults. Age correlated negatively with all cognitive variables. Specifically, negative correlations were observed with verbal WM measures, consistent with previous studies (e.g., Bopp and Verhaeghen, 2005; Borella et al., 2008; Rhodes and Katz, 2017; Sander et al., 2012). Measures of processing speed and crystallized intelligence also correlated negatively with age, with the correlation for processing speed (−0.63) being stronger than that for crystallized intelligence (−0.37). Similar findings were reported by Baltes and Lindenberger (1997) in a cross-sectional sample of 516 adults aged 70–103 years, where perceptual speed showed the strongest negative association with age (*r* = −0.59) and knowledge the weakest (*r* = −0.42). The negative correlation between age and tests of crystallized abilities may reflect a cohort effect, in that older adults may have had fewer opportunities for formal education, resulting in lower vocabulary scores. However, longitudinal studies have demonstrated that crystallized abilities (e.g., vocabulary) decline after the age of 60 (Rönnlund et al., 2005; Schaie, 2005). Furthermore, a meta-analysis reported a negative correlation between age and vocabulary in samples over 60 years of age (Verhaeghen, 2003). Strong positive correlations were observed among processing speed, crystallized abilities, and verbal WM performance indices. This pattern suggests substantial shared variance among these cognitive constructs, potentially reflecting common underlying cognitive resources (Lindenberger, 2001; Li et al., 2004).

Two moderate mediation analyses were conducted. Both crystallized abilities (first analysis) and processing speed (second analysis) were significant mediators of the relationship between age and verbal WM such that higher scores in these domains predicted better verbal WM performance. However, neither crystallized abilities nor processing speed interacted significantly with age. These findings suggest preserved differentiation, that is, stable individual differences in cognitive functioning (i.e., crystallized abilities and processing speed) rather than differential rates of age-related decline (Salthouse, 2006; Tucker-Drob et al., 2009).

In the first analysis, processing speed significantly moderated the path between age and crystallized intelligence, yielding a conditional indirect effect of age on verbal working memory among participants with high processing speed. This suggests that individuals with higher processing speed maintain higher crystallized abilities into advanced age, whereas those with average or low processing speed do not benefit and may even show declines. Thus, high processing speed appears to support the maintenance of crystallized abilities in older age. Depressive symptoms exerted a marginally significant influence on WM performance but did not alter the strength or direction of the effects among age, crystallized abilities, and processing speed.

In the second analysis, the moderated mediation index was not significant when crystallized abilities were included as a moderator. This indicates that crystallized abilities did not exert an indirect effect via processing speed. In other words, while age-related changes in processing speed predicted crystallized abilities, the reverse was not true. These results align with Ghisletta and Lindenberger (2003), who showed that longitudinal changes in crystallized abilities (e.g., vocabulary knowledge) are better predicted by processing speed than vice versa.

Taken together, the results suggest that processing speed and crystallized knowledge jointly influence verbal WM performance. Older adults who preserve higher levels of fluid abilities (e.g., processing speed) benefit more from crystallized abilities, which in turn enhances working memory. Conversely, greater losses in fluid abilities reduce the potential benefits of crystallized abilities, leading to poorer verbal WM performance. These findings are consistent with Tucker-Drob et al. (2022), who demonstrated strong correlations between longitudinal changes in fluid and crystallized abilities.

Overall, the results of this study indicate limits to the extent that crystallized abilities can compensate for age-related declines in WM. Only individuals with high processing speed appear able to maintain crystallized intelligence at levels that support WM. From a cognitive training perspective, these results may clarify the conditions under which interventions are effective (Takeuchi and Kawashima, 2012). Faster processing speed enables older adults to encode, integrate, and retrieve information more efficiently, thereby facilitating the acquisition of new vocabulary and knowledge. Consequently, interventions aimed at enhancing processing speed may increase learning capacity and create the conditions necessary for the continued acquisition and use of crystallized knowledge. Research has shown that improvements in processing speed among older adults are associated with greater vocabulary acquisition and overall cognitive performance (Ball et al., 2007; Bonnechère et al., 2021). Furthermore, cognitive training programs focused on processing speed have demonstrated transferable benefits to untrained tasks, suggesting that these interventions can have broad effects on everyday cognitive functioning (von Bastian et al., 2022) and individuals with lower processing speed may be those who benefit most from such training (Schiff et al., 2021).

Limitations of this study include the cross-sectional design, which precludes causal inference, and potential cohort effects. Future research should employ longitudinal designs to examine intraindividual change and the interplay between fluid and crystallized abilities over time. Furthermore, the sample has a higher number of women than men, which could limit the generalizability of the results to the male population.

In conclusion, this study highlights the interactive roles of processing speed and crystallized abilities in age-related WM decline and suggests that maintaining processing speed may be key to supporting cognitive performance in older adulthood.

## Data Availability

The raw data supporting the conclusions of this article will be made available by the author, without undue reservation.
